# Evaluating the Efficacy of Ethical Guidelines for Online Screening of Mental Health in South Africa

**DOI:** 10.3389/fpsyg.2022.875911

**Published:** 2022-07-14

**Authors:** Tasneem Hassem, Sumaya Laher

**Affiliations:** Department of Psychology, University of the Witwatersrand, Johannesburg, Johannesburg, South Africa

**Keywords:** AGREE, ethics, mental health, online screening, telehealth

## Abstract

Despite the rapid growth in digital mental health options, a systematic review conducted on the ethics of developing online mental health screening instruments highlighted that there were no formal guidelines in this area. This lack of formal guidelines and the results of the systematic review led to the development of formal guidelines for online mental health screening tools in South Africa. This study aimed to explore the efficacy of these draft guidelines using a qualitative design with two samples of individuals recognised as experts in the field of mental health. Sample one consisted of a purposive sample of 15 experts who commented on the appropriateness of the draft guidelines. The second sample consisted of 9 experts who completed the Appraisal of Guidelines for Research and Evaluation (AGREE) instrument to assess the guidelines. Both samples were in agreement on the relevance of the core content areas in the guidelines, namely purpose and scope, modes of testing, psychometric properties, informed consent, ensuring minimal risk to participants, feedback as well as data security. There was also agreement on the appropriateness of the methods used to develop the guidelines. The use of the guidelines was supported with the suggestion that issues of risk and suicidality be explored further.

## Introduction

Globally mental health disorders have been increasing more so since the advent of the COVID-19 pandemic (Rajkumar, 2020; Zhou et al., 2020). Recent studies have shown an increase in psychological symptoms of distress amongst the general population across the world, with a particular increase in anxiety and depression rates (Rajkumar, 2020; Salari et al., 2020). South Africa evidences similar mental health trends amongst the population. According to the South African Stress and Health (SASH) study, 30.3% of South Africans are likely to suffer from a mental disorder in their lifetime (Herman et al., 2009). At the time of the SASH study, South Africa reported a 12-month prevalence of 16.5% for mental disorders (Williams et al., 2008). Despite the burden of mental illness, mental health facilities in South Africa are often under resourced with general hospitals providing 2.8 beds per 100,000 population, community facilities accommodating 3.6 beds per 100,000 and mental health hospitals accommodating 18 beds per 100,000. In addition, these facilities are also under staffed resulting in the underdiagnosis and treatment of mental health disorders (Lund et al., 2010, 2012; Burns, 2011; Matsea et al., 2018). There is an urgent need for resources to aid with screening, diagnosis and intervention for mental health difficulties in the population.

Cortelyou-Ward et al. (2018) amongst others recommended using technology to assist with access to appropriate mental health care. “Telehealth” is the term given to encompass all healthcare including mental health care provided through the use of various technologies, which are inclusive but not necessarily limited to the internet (Joint Task Force for the Development of Telepsychology Guidelines for Psychologists, 2013; Zhou et al., 2020). These activities include: screening, assessment, diagnosis, intervention, psychoeducation and support amongst others and can involve purely virtual input (no direct contact with a person) or face to face input using virtual conferencing or similar engagement (Zhou et al., 2020). The use of telepsychology in the South African context is fairly limited. Goldschmidt et al. (2021) argue that this could be a result of the high data costs in the country as well as the lack of training on the use of telepsychology. However, the context of the pandemic has ensured a rapid switch to telepsychology with very few guidelines for practitioners on utilising psychotherapy and psychological assessment in this space. August and Mashegoane (2021) reported that telepsychology guidelines emanating from African organisations such as the Health Professions Council of South Africa (Health Professions Council of South Africa, 2020) and international guidelines from the British Psychological Society (British Psychological Society, 2021) and the American Psychological Association (American Psychological Association, 2020) have been generic, presenting overarching factors that practitioners should consider when conducting assessments. Additionally in the South African context, Evans (2018) and Chipise et al. (2019) also provide brief guidelines for telepsychology. Psychological assessment is briefly mentioned in each of these documents, but the focus is more on guidelines for practitioners using tests online. There are currently no psychometrically validated, online screening instruments for mental health developed in South Africa with the exception of an online, open access screening tool for depression (see Hassem, 2021). Using psychological assessment in the online space introduces a myriad of ethical concerns from issues around confidentiality through to risk and data security necessitating ethical guidelines.

Ethics typically refers to “a set of certain, aspirational moral values and principles that are intended to guide ethical conduct” (Walsh, 2015, p. 69). In the field of psychology the need for ethical guidelines can be traced back to the World Wars and the tests used with soldiers as well as the many soldiers returning from war with mental health difficulties. Indeed this led to ethical standards in the health sciences more broadly. The American Psychological Association (APA) developed its first formal ethical code in 1953. This code was revised thrice and currently speaks to Beneficence and Non-maleficence, Fidelity and Responsibility, Integrity, Justice, and Respect for People’s Rights and Dignity (Walsh, 2015).

In South Africa, it was clear as early as the 1920’s that gross ethical violations were occurring within psychology particularly within psychological assessment (Fick, 1929). Systematic discrimination on the basis of skin colour was the norm in South Africa prior to 1994. Individuals of European origin (White) people were privileged over Black (indigenous populations living in South Africa as well as all immigrants not of European descent) people. Hence tests developed in Europe and the United States were used with Black people (Fick, 1939). When Black people performed poorly, it was concluded that this was due to genetically lowered ability. Such gross misconduct led to a huge distrust in psychological assessment until South Africa became a democracy in 1994 (Laher and Cockcroft, 2014). In 2006, the Professional Board for Psychology at the Health Professions Council of South Africa promulgated its ethical code for psychologists (Government Gazette, 2006). Chapter 5 of the code addresses ethical issues linked to psychological assessment.

**FIGURE 1:**
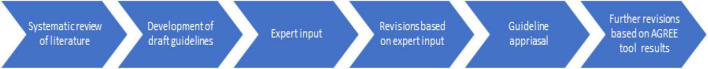
Guideline development process.

Aside from South Africa and the United States, various ethical documents have been developed detailing the specific ethical criteria which need to be adhered to by the professionals involved in online psychological consulting (Fisher and Fried, 2003; International Test Commission, 2005; Joint Task Force for the Development of Telepsychology Guidelines for Psychologists, 2013; Luxton et al., 2016; Evans, 2018). However, these are primarily focussed on therapeutic interventions and provide minimal information with regards to the ethical concerns that might be necessary for online mental health screening Therefore, while a strong need exists for online tools that can aid screening or diagnosis of mental health, there are no clear guidelines with regards to the ethical considerations for such an undertaking (see Hassem and Laher, 2020). Using the recommendations provided by Hassem and Laher (2020), a draft guidelines document was developed as a supplementary document to various international best practice guidelines documents like the International Test Commission (ITC) Guidelines for Translating and Adapting Tests – Second Edition (International Test Commission, 2017), the ITC Guidelines on Test Use (International Test Commission, 2013), and the ITC Guidelines on Computer-Based and Internet Delivered Testing (International Test Commission, 2013). The stages involved in the development of the ethical guidelines for online screening instruments are represented in Figure 1.

The draft ethical guidelines document aims to provide guidelines regarding the ethical considerations for all stakeholders involved in the development and placement of mental health screening tools on a digital platform. The document specifically addresses ethical concerns regarding open mode tests with regards to purpose and scope, modes of testing, psychometric properties, informed consent, ensuring minimal risk to participants, feedback as well as data security. Table 1 provides a brief overview of the sections in the guidelines document.

**TABLE 1:** Overview of the guidelines document.

1. Introduction	6. Acknowledgements
2. Aim	7. Competing interest
3. Objectives	8. Funding
4. Who are the guidelines for?	9. Disclaimer
5. Process of guideline development:	10. Guidelines for developing an open mode online mental health screening tool
5.1. Systematic Review	10.1. Purpose or scope
5.2. Development of draft guidelines	10.2. Modes of testing
5.3. Expert input	10.3. Psychometric properties
5.4. Revision	10.4. Informed consent
5.5. Guideline appraisal	10.5. Ensuring minimal risk to participants
5. 6. Revisions	10.6. Feedback
5.7. Procedures for updating the guidelines	10.7. Data security
	11. References

Within the digital space, documents like the ethical guidelines can contribute to ensuring individual wellbeing and empowering individuals to be proactive in ensuring their own mental health. Documents like these serve to facilitate the creation of online wellness spaces in the current climate where online mental health is fast becoming a norm. Additionally, the guidelines serve to support professionals working in the field as a point of reference for designing as well as assessing the quality of online screening instruments. This is more so for contexts like South Africa where access to mental health resources are lacking but also where assessment tools are not always applicable to the majority of the population. For us the development of guidelines was paramount as we had embarked on a project to develop an open access online screening tool for depression for use with the South African population (Hassem, 2021). Whilst doing this we were unable to find a comprehensive document that spoke to the ethics of online screening of mental health. Hence the systematic review of literature and the development of the draft guidelines for the online screening of mental health instruments (Hassem and Laher, 2020).

This study aimed to explore the efficacy of the draft guidelines by consulting with experts in the field with the objective of producing an open access ethics guidelines document for use in developing and adapting online screening tools for mental health.

## Materials and Methods

This study utilised a qualitative research design where two samples of experts in the field of psychology and psychiatry were invited to review each section of the draft ethical guidelines for online mental health screening. After feedback was received from Sample 1, the draft guidelines were updated based on the expert feedback received and a second group of experts were asked to comment on the guidelines using the Appraisal of Guidelines for Research and Evaluation (AGREE) instrument (Brouwers et al., 2010). Hence the approach with sample 1 was inductive where the themes extracted were data driven whilst the approach with sample 2 was deductive with themes reported as per the AGREE domains (Braun and Clarke, 2006). The design and reporting of the study conforms with the reporting standards for qualitative research as proposed by Levitt et al. (2018).

## Sample 1

A purposive sample of 50 international and South African experts in the field of psychology or cognate disciplines like psychiatry and who had experience in psychometrics, test development and/or ethics were invited to participate in the study. We identified experts to approach via their publications or presentations at local and international conferences. A total of 15 experts responded to the invitation. Of the 15 experts, eight identified as psychologists, six as psychiatrists, and one as a mental health activist. Majority of the sample (*n* = 8) were male and South African (*n* = 12). Only twelve experts stated their age, with the majority falling in the 50–60 years age category, while one expert was in the 60+ category. Majority of the sample (*n* = 5) were practising in the field between 11 and 20 years, while two experts were practising between 30 and 40 years.

### Instruments

Participants were required to complete a brief demographic questionnaire that requested information regarding gender, age, occupation and experience. Participants were also provided with the draft ethics guidelines document. The ethics guideline document provided a glossary of key terms used in the document, followed by 5 specific sections which spoke to: purpose or scope, modes of testing, psychometric properties, informed consent, feedback and data security. Two open ended questions were asked of the experts regarding the appropriateness of the guidelines as well as recommendations for amendments to be made to the document based on their expertise.

### Procedure

The study was approved by the Human Research Ethics Committee, Medical (HRECM) of the University of the Witwatersrand, Johannesburg (Ethics protocol number: M180402). Ethical guidelines were developed from a systematic review conducted on ethics of online screening for mental health in South Africa (Hassem and Laher, 2020). Once the guidelines were developed, they were emailed to potential participants. Participants were asked to review the guidelines document and provide feedback on the efficacy of the guidelines.

### Data Analysis

The demographic data was analysed using frequencies while all feedback was analysed using thematic analysis. Both of us were involved in the coding process and we utilised an inductive, latent approach in order to determine themes as recommended by Braun and Clarke (2006). We are both registered as research psychologists with the HPCSA in South Africa and we both have some counselling experience with individuals who have been diagnosed with depression. Additionally we both have experience in psychometrics and test development. We followed the six steps recommended by Braun and Clarke (2006) to conduct the thematic analysis. This involved familiarisation and coding of extracted data, followed by theme development.

### Results

Based on the analysis of the results, five main themes were evident: appraisal of the guidelines, informed consent, experience of distress, feedback and confidentiality. Minor editorial changes for language, grammar, and typographical edits were not included as a theme. These were corrected in the document.

#### Appraisal of the Guidelines

Majority of the experts (*n* = 9) felt that the draft guidelines provided were useful and much needed. Participant 11 represented this well with: “they give structure and shape the effort, especially for less experienced researchers/practitioners in this field. They certainly offer a check-list against which to compare the initial blueprint of the development process, or the results.”

#### Informed Consent

Five participants suggested that the manner in which the data provided by the end-users and how the results of an online mental health screening tool is utilised in terms of research purposes as well as the company hosting the website needed to be stated. Participant 3 best expressed this as “what about using the data for research and how the results will be used by the organisation hosting the website?” In addition, one participant (P1) noted that under the informed consent section in the guidelines document, it would be necessary to include that end-users should consent to the potential distress that could be experienced as well as the responsibility for self-help seeking behaviour.

#### Experience of Distress

Despite not having a specific section dedicated to experience of distress, four participants felt that warnings or red flags should appear prior to a potentially distressing question being asked. Participants also felt that crisis assistance should be provided on every page of the screening tool as distress could occur at any point in time. Participant 2 commented “contact numbers of support services should appear PROMINENTLY on every screen so that at any point when the person feels overwhelmed, they will know who to contact.”

#### Feedback

Ten out of the 15 experts felt that the feedback section needed to be further developed. Participants mentioned that a specific statement needed to be made that the feedback provided is written up using language and tone that would encourage the end-user to seek help. This is captured in the comment provided by participant 7 “The language, tone and indicators are all critical in ensuring referral uptake.” In addition, the feedback should also address possible issues regarding stigmatisation of mental illness and highlight that mental illness is treatable. Lastly, participants felt that the inclusion of tracking individuals who are a threat to themselves would not be realistic as locations obtained from mobile devices are constantly changing and this would infringe on anonymity (website locations) and informed consent. This view is further highlighted by the following comment: “consider those using mobile devices, you will not receive an exact location. Once you start tracking individuals, you need their consent and there are a number of ISO standards with which you need to comply to protect their privacy” (Participant 7).

#### Confidentiality

Confidentiality was raised by 7 participants in terms of data security as these two concepts cannot be separated. Five experts felt that the data stored on the server that has routine backups in place could pose a risk to confidentiality and therefore alluded to the fact that data should be coded. The concern around confidentiality is evident in the statement made by participant 13: “The issue of Confidentiality is of great concern EVEN IF THE TOOL IS ANONYMOUS. There needs to be an insertion concerning confidentiality. If the information is sent to a server, it may be accessible to more than one person.” In addition, two participants suggested that the General Data Protection Regulation documents of data security as applicable to the country/ies the screening tool would be used in, should be consulted.

## Sample 2

A purposive sample of 31 international and South African experts in the field of assessment were approached to participate in the study. Nine experts responded to the call. Majority of sample were female (*n* = 8) and from South Africa (*n* = 8). All participants identified as being psychologists, with the majority identifying as research psychologists. It is important to note that in South Africa test development falls under the scope of practice of the research psychologist with those registering in other categories like clinical, counselling or educational psychology receiving little or no training in this area. Hence experts would largely hold a research psychology and/or psychometry registration. Majority of the experts were in the 45–65 years age range. Five experts were practicing in excess of 25 years, 2 experts in excess of 10 years and 2 experts were practicing between 2 and 5 years.

### Instruments

The AGREE checklist was developed by AGREE Next steps Consortium to ensure the quality of practise guidelines AGREE Next Steps Consortium, 2017. The checklist consists of 23 questions which assess the guidelines in the health field based on six broad domains; scope and purpose, stakeholder involvement, rigour of development, clarity of presentation, applicability and editorial independence (Brouwers et al., 2010). The 23 questions follow a Likert type scale, where 1 represents strongly disagree and 7 strongly agree. We added a *Not applicable* option to the scale as this checklist is used specifically in assessing practise guidelines in the health sector and not for appraisal of documents like ethical guidelines. Each question had a comment section after the rating, where participants could elaborate on the score provided if they so wished. Two additional questions assessed the overall guidelines in terms of quality rating, the first question asked participants to rate the guidelines on a scale of 1–7, with 1 representing lowest possible quality and 7 highest possible quality. The final question asked participants how likely they were to recommend the use of the guidelines with the following response options: yes, yes with modification and no.

### Procedure

Feedback from Sample 1 was used to refine the ethical guidelines. Following this, the revised guidelines were emailed to potential participants. Participants were asked to assess the guidelines as per the categories in the AGREE instrument.

### Data Analysis

Scores on the AGREE tool were not calculated as per the user manual as the tool needed to be adapted to suit the categories against which the screening tool was developed. Hence totals were not calculated for each section as the inclusion of a not applicable section would not provide an accurate reflection for each section if it was totalled. Frequencies for the responses on each of the AGREE items were considered. In addition, the comments provided in the open ended section of each item as well as any suggested changes made on the actual guideline document were noted. Thus the analysis proceeded deductively using content analysis and semantic themes (Braun and Clarke, 2006).

### Results

**FIGURE 2:**
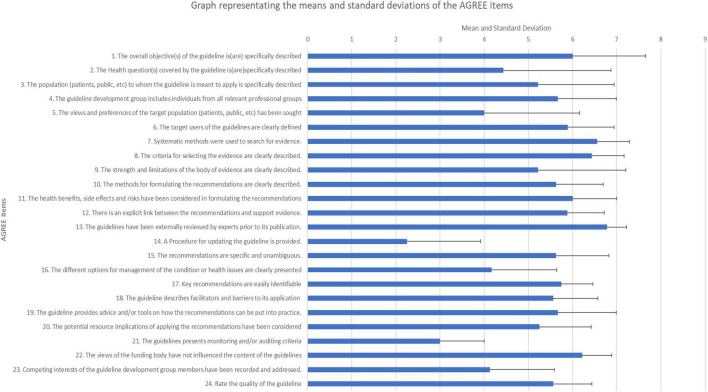
A graphical representation of the means and standard deviations for each AGREE item.

Results for each of the 6 content domains of the AGREE are presented in Table 2 in terms of frequencies. Figure 2 presents a graphical presentation of the means and standard deviations of item. Results are discussed along with the qualitative feedback under the relevant headings below.

**TABLE 2:** Frequency responses obtained on the AGREE tool.

Item	1*	2	3	4	5	6	7*	N/A
**Domain 1: Purpose and scope**								
1. The overall objective(s) of the guideline is(are) specifically described		1			1	2	5	
2. The Health question(s) covered by the guideline is(are)specifically described	2				2	2	1	2
3. The population (patients, public, etc.) to whom the guideline is meant to apply is specifically described		1	1		2	3	2	
**Domain 2: Stakeholder involvement**								
4. The guideline development group includes individuals from all relevant professional groups				2	3		4	
5. The views and preferences of the target population (patients, public, etc.) has been sought	1	1	1	1	1	1	1	2
6. The target users of the guidelines are clearly defined				1	2	3	3	
**Domain 3: Rigour of development**								
7. Systematic methods were used to search for evidence					1	2	6	
8. The criteria for selecting the evidence are clearly described					1	3	5	
9. The strength and limitations of the body of evidence are clearly described	1		1		1	4	2	
10. The methods for formulating the recommendations are clearly described				1	3	2	2	1
11. The health benefits, side effects and risks have been considered in formulating the recommendations					3	1	3	2
12. There is an explicit link between the recommendations and support evidence					3	3	2	1
13. The guidelines have been externally reviewed by experts prior to its publication						2	7	
14. A Procedure for updating the guideline is provided	3	3	1			1		1
**Domain 4: Clarity of presentation**								
15. The recommendations are specific and unambiguous				2	1	3	2	1
16. The different options for management of the condition or health issues are clearly presented		1	1	1	2	1		3
17. Key recommendations are easily identifiable					3	4	1	1
**Domain 5: Applicability**								
18. The guideline describes facilitators and barriers to its application				1	4	2	2	
19. The guideline provides advice and/or tools on how the recommendations can be put into practice			1		3	2	3	
20. The potential resource implications of applying the recommendations have been considered			1		4	2	1	1
21. The guidelines presents monitoring and/or auditing criteria		2	4		1			2
**Domain 6: Editorial independence**								
22. The views of the funding body have not influenced the content of the guidelines					1	5	3	
23. Competing interests of the guideline development group members have been recorded and addressed.	1			4	2	1		1
**Overall guideline assessment**								
24. Rate the quality of the guideline				1	3	4	1	
25. I would recommend this guideline for use	Yes (*n* = 6)		Yes, with modification (*n* = 3)				No (*n* = 0)	

*1 represents strongly disagree and 7 strongly agree.

#### Domain 1: Scope and Purpose

Item 1 in this domain asked whether the objectives of the guidelines had been specifically described. Majority of the experts (*n* = 5) gave a rating of 7, with the item receiving a mean score of 6 (SD = 1.66) and scores ranging from 2 to 7. Despite the overall high rating on this item, there were two experts who indicated that while the guidelines provided an aim, a specific section titled objectives needed to be added as noted by participant 8 “there is no specific section that pertains to the objectives of the guidelines, only a small section on the aim of the guideline. Specific objectives should be elaborated on”

With regards to the second item in this domain that asked whether the health questions that guidelines pertained to were specifically stated, majority (*n* = 5) of the experts rated this item above 5, with a mean score of 4.43 (SD = 2.44) and scores ranging from 1 to 7, with 2 experts arguing this item was not applicable as ethics guidelines do not necessarily speak to a health domain, rather these are ethical guidelines within the field of psychometry and instrument development.

Item three under domain 1 asked if the population to whom the guidelines are meant to apply was clearly delineated. This item received a rating of 5 or above from majority (*n* = 7) of the experts, with a mean score of 5.22 (SD = 1.72) and scores ranging from 2 to 7. Experts did indicate that this section should be more explicit in the guidelines.

#### Domain 2: Stakeholder Involvement

The first item in this domain assessed the representation of professionals involved in the development of the guidelines (item 4) and received a maximum score of 7 by 4 experts, with a mean score of 5.67 (SD = 1.32) and scores ranging from 4 to 7. Based on the elaborated responses, experts indicated that more details needed to be provided about the individuals involved in the guideline development phases in terms of their professions. This is captured by the comment made by participant 2: “would be useful to indicate who the professional group are. Psychologist, social workers psychiatrist?”

The second item in this domain (item 5) dealt with obtaining views from the target population on the screening. This item was generally well received with a mean score of 4 (SD = 2.16) and scores ranging from 1 to 7. Two individuals found the item as being not applicable to ethics guidelines.

The last item in this domain (item 6) assessed the clarity in defining the target users of the guidelines. Majority of the experts (*n* = 8) rated this item as a 5 or above with a mean score of 5.89 (SD = 1.05) and scores ranging from 4 to 7. Experts suggested that this item would benefit from more specific information on the target users of the guidelines being provided.

#### Domain 3: Rigour of Development

Majority of the experts (*n* = 6) felt that a rigorous process was followed to develop the guidelines (item 7), with a mean score of 6.65 (SD = 0.73) and scores ranging from 5 to 7. This is best indicated by Participant 3: “a thorough literature review was conducted and a peer reviewed paper has been written outlining these results.”

Item 8 which asked whether a clear description for selecting evidence received was provided. This item received a response of 7 from majority of experts (*n* = 5), with a mean score of 6.44 (SD = 0.73) and scores ranging from 5 to 7. With regards to the strengths and limitations of the body of the evidence (item 9), majority of the experts rated this item with a 5 or above, with a mean score of 5.22 (SD = 1.99) and scores ranging from 1 to 7 indicating that experts found the development process rigorous and evidence-based.

Majority of experts rated items 10 (methods for developing guidelines are clearly described) (*M* = 5.63, SD = 1.06), 11 (health benefits, side effects and risks have been considered in formulating the recommendations) (*M* = 6, SD = 1) and 12 (there is an explicit link between the recommendations and support evidence) (*M* = 5.88, SD = 0.84) with a 5 or higher. The link between the recommendations and support evidence is highlighted by the comment made by participant 4, “As evidenced by how the whole document is argued and structured.”

Item 13 (guidelines have been externally reviewed by experts prior to its publication) was rated 7 by the majority of experts (*n* = 7) with a mean of 6.78 (SD = 0.44) and scores ranging from 6 to 7. The high mean score on this item is echoed in the following comment (P4), “As is clear from the actions taken while drafting the first version, and now again in consulting more reviewers pertaining to the current version. National and international experts were included.” Lastly, majority of the experts (*n* = 7) felt that a procedure for updating the guidelines was not clearly provided (*M* = 2.25, SD = 1.68) as noted by participant 3, “This should be more explicit.”

#### Domain 4: Clarity of Presentation

Majority of the experts (*n* = 6) (*M* = 5.63, SD = 1.19) felt that the recommendations were specific and unambiguous as indicated by ratings of 5 or higher. Item 16 which asked about different options for management of the condition or health issues received varied responses, with 3 experts rating the item as not applicable (*M* = 4.17, SD = 1.47) with participant 4 providing the following comment: “Accomplished through feedback to the client, and further referrals for support to psychological services (e.g., Guideline section “Feedback”).” Majority of experts (*n* = 8) rated item 17 (Key recommendations are easily identifiable) as a 5 or above with a mean score of 5.57 (SD = 0.71) with scores ranging from 5 to 7.

#### Domain 5: Applicability

The first two items on this domain which assessed the barriers and facilitators (The guideline describes facilitators and barriers to its application) and advice on recommendation implementation (The guideline provides advice and/or tools on how the recommendations can be put into practice) were rated a 5 or above by the majority of experts (*n* = 8) (Item 18, *M* = 5.56, SD = 1.01; Item 19, *M* = 5.67, SD = 1.32). Participant 4 provides a description of where these barriers and recommendations were made in the document, “As accomplished by all of the Guidelines’ parts on data security (see Supplementary section 7), feedback and referral procedures (see Supplementary section 6), minimising risk (see Supplementary section 5), informed consent (see Supplementary section 4), and psychometric properties (see Supplementary section 3).”

Item 20 (potential resource implications of applying the recommendations have been considered) (*M* = 5.25, SD = 1.17) was well received by participants with 7 experts providing a rating of 5 or higher. Participant 6 provided further suggestions for consideration, “Perhaps consideration can be given to ethics re. bandwidth, connectivity and device issues that may present during screening, e.g., allowing a user to complete the tool more than once, in case they get kicked out For item 21 (presents monitoring and/or auditing criteria), a fairly low rating was achieved, with 4 experts providing a rating of 3 (*M* = 3, SD = 1). The low rating provided can be attributed to the question not being applicable to the guidelines as indicated by 2 experts and evidenced in the following comment made by participant 8: “Not sure if this is relevant, or if such a mechanism will have to be lodged somewhere?”

#### Domain 6: Editorial Independence

Item 22 (views of the funding body have not influenced the content of the guidelines) received ratings that ranged from 5 to 7 with majority of experts (*n* = 5) providing a rating of 6 (*M* = 6.22, SD = 0.67). Experts noted, however, that there should be a clear and explicit statement about funders in the guidelines. With regards to item 23 (competing interests of the guideline development group members have been recorded) a majority rating of 4 (*n* = 4) (*M* = 4.13, SD = 1.46) and one not applicable rating was received with an expert feeling that competing interests for the professionals involved in the development of the guidelines were not immediately obvious and needed to be stated explicitly.

#### Overall Guideline Assessment

Majority of experts rated the overall quality of the guidelines with a 5 or higher (*n* = 8), with 6 experts recommending the use of these guidelines without modifications. Three experts felt the guidelines needed modifications as per the feedback provided on each of the AGREE tool items. This is echoed in the following comment made by participant 2: “I found the recommendations to be the strength of the document. It is well thought out, researched and put together. The first part of the document requires some more clarity. This is a very much needed document in the clinical environment where screening tools are used all the time.”

## Discussion

This study set out to revise the draft of ethical guidelines on the online screening of mental health as developed from the results of a systematic review by Hassem and Laher (2020). The study used two samples of experts to review the draft guidelines. After the feedback from the first sample, the guidelines were revised and presented to a second sample of experts who rated the guidelines according to the AGREE tool – a measure that was developed to objectively assess the quality of guidelines in health fields. The results from the AGREE tool were encouraging with majority of the experts supporting the use and dissemination of the guidelines. However, under each of the 6 domains of the AGREE tool, experts provided suggestions for improving the clarity of the guidelines. Based on the feedback provided the guidelines were revised to include a section on objectives, more explicit information on the target population as well as target users, explicit statements on updating the guidelines, independence from funding bodies and a declaration of no competing interests. These amended guidelines are provided in Supplementary Appendix A.

It should be noted that the items assessing the rigour of the document received the highest rating with majority of the experts indicating that the methods to develop the guidelines were rigorous and clear. Of further noting is that none of the experts in the second sample raised issues relating to the content of the guidelines. Some provided feedback on issues of language and grammar but there was consensus that the actual categories and recommended guidelines within each of the categories were relevant and useful.

This document comes at a time when individuals are highly prone to mental health disorders as a result of the COVID-19 pandemic, and mental health professionals are charting a relatively new landscape. This contribution of a formal ethical guideline document for online mental health screening makes a novel contribution to knowledge in the global and local space. It is to our knowledge the first document, both globally and locally, dedicated to addressing the ethical concerns of developing and placing an open mode psychological screening test in the online environment. The document provides practical implementation guidelines which can be used to foster positive ethical behaviour in the online environment by encouraging developers to consider issues relating to access, fairness, minimal harm and data protection. In so doing the guidelines also contribute to a social justice imperative.

Anecdotal evidence on the efficacy of the guidelines comes from Hassem (2021, 2022), where these guidelines were followed in creating an open access depression screening tool and subsequently placing the tool on a website for the online screening of depression^1^. The guidelines informed the adaptation of the CESD-R tool^2^ by providing some direction on items to include online given the unsupervised nature of online screening as well as considerations for the accessibility and fairness of the screening tool. For example, all items linked to suicide and suicidal ideation were removed from the tool. Given the limited resources and mental health infrastructure in South Africa, it would be unethical to assess suicide ideation when the country does not have designated resources available to contact the individuals who display suicide ideation tendencies. The inclusion of this domain can only happen once resources are available in South Africa that allow efficient and effective tracking of individuals who endorse these items during the online screening. In addition, in order to ensure accessibility and fairness, the language used excluded any form of psychological jargon and included South African idioms of distress. Whilst it may be argued from a clinical perspective that suicidal ideation is vital for depression screening and diagnosis, the nature of online unsupervised screening suggests a risk that would outweigh the benefits more so in light of the limited resources available for support in South Africa. The website design was also informed by the ethical guidelines to ensure accessibility (minimal images), fairness (simple, easy to understand English used, inclusive of local idioms of distress), minimal harm (contact details of various organisations are provided which are inclusive of toll-free numbers) and data protection (routine monitoring and updates of the site; see Hassem, 2021).

Whilst these guidelines have demonstrated utility based on feedback from two samples of experts as well as through a project on the online screening of depression in South Africa, more research is required to finalise the guidelines. Despite the useful data obtained from both samples, the samples were small with low response rates. No doubt the context of the pandemic in 2021 contributed to this as people locally and internationally had less time for this type of work. Going forward it is necessary to adopt a more quantitative, cross sectional approach and approach a larger, more diverse sample to rate the guidelines using the AGREE tool and to provide further commentary on refining and finalising the guidelines. In particular issues of beneficence and maleficence need to be addressed especially as they pertain to self-harm and risk. Excluding items on suicide or suicidal ideation, for example, is a major limitation but ensuring care post screening is also an ethical responsibility. The guidelines should provide more direction for negotiating such dilemmas. Additionally, it is necessary to compare these guidelines against the set of principles in the *Universal Declaration of Ethical Principles for Psychologists* (International Union of Psychological Science, 2008). Anecdotally the guidelines conform to the principles but a more systematic exploration of this would be useful.

## Conclusion

The online screening of mental health is often suggested as a means of ensuring access to healthcare services for those who may not be able to consult with a professional. However, it was clear from the literature that there were no ethical guidelines for the online screening of mental health. Hence a set of guidelines was developed based on the findings of Hassem and Laher (2020). This study used two sets of experts to evaluate these draft guidelines. The experts agreed that the guidelines are much needed in the field of online screening of mental health. Based on the feedback received, the draft guidelines were revised and are appended to this article (see Supplementary Material). It is hoped that these draft guidelines will assist others in ensuring that ethical and rigorous procedures are followed in the development, adaptation and use of online screening instruments for mental health. Going forward the aim is to finalise the guidelines for use internationally to ensure best practice in the online screening of mental health.

## Data Availability Statement

The original contributions presented in this study are available from the authors upon request.

## Ethics Statement

Ethical clearance for this study was obtained from the Human Research Ethics Committee, Medical (HRECM) of the University of the Witwatersrand, Johannesburg (Ethics protocol number: M180402). The patients/participants provided their written informed consent to participate in this study.

## Author Contributions

Both authors involved in conceptualising the guidelines, and contributed to data collection, results, analysis, and write-up of the manuscript and guidelines.

## Conflict of Interest

The authors declare that the research was conducted in the absence of any commercial or financial relationships that could be construed as a potential conflict of interest.

## Publisher’s Note

All claims expressed in this article are solely those of the authors and do not necessarily represent those of their affiliated organizations, or those of the publisher, the editors and the reviewers. Any product that may be evaluated in this article, or claim that may be made by its manufacturer, is not guaranteed or endorsed by the publisher.

## Footnotes

1
www.mddsa.co.za

2
https://cesd-r.com
