# Supplementary material for: Evaluating the Efficacy of Ethical Guidelines for Online Screening of Mental Health in South Africa
Source: Front Psychol. 2022 Jul 14;13:875911. doi: 10.3389/fpsyg.2022.875911 (PMC9331289; doi:10.3389/fpsyg.2022.875911)
Supplement: Supplementary file 1 [file Data_Sheet_1.docx]

**Appendix A** - Ethical Guidelines for Developing an Online Mental Health Screening Instrument

**These guidelines were developed in South Africa by** Dr Tasneem Hassem & Professor Sumaya Laher from the Psychology Department at the University of the Witwatersrand, South Africa**. In order to use these guidelines in another country, it is recommended that in addition to the international best practice documentation, the relevant legislation or other documented guidelines of the particular country be considered.**

**How to cite these guidelines**

Hassem, T., & Laher, S. (2020). *Ethical guidelines for developing an online mental health screening instrument.* The University of Witwatersrand, Johannesburg.

For comments or recommendations on enhancing these guidelines email [Tasneen.hassem@wits.ac.za](mailto:Tasneen.hassem@wits.ac.za) or [Sumaya.Laher@wits.ac.za](mailto:Sumaya.Laher@wits.ac.za)

**Contents**

**Introduction** [21](#_heading=h.3znysh7)

**Aim** [21](#_heading=h.2et92p0)

**Objectives** [21](#_heading=h.tyjcwt)

**Who are the guidelines for?** [22](#_heading=h.3dy6vkm)

**Process of Guideline development** [22](#_heading=h.1t3h5sf)

**1.** **Systematic review** [22](#_heading=h.4d34og8)

**2.** **Development of draft guidelines** [23](#_heading=h.2s8eyo1)

**3.** **Expert input** [24](#_heading=h.17dp8vu)

**4.** **Revision** [24](#_heading=h.3rdcrjn)

**5.** **Guideline appraisal** [24](#_heading=h.26in1rg)

**6.** **Revisions** [25](#_heading=h.lnxbz9)

**7.** **Procedures for updating the guidelines** [25](#_heading=h.35nkun2)

**8. Acknowledgements** [25](#_heading=h.1ksv4uv)

**9. Competing interests** [26](#_heading=h.44sinio)

**10. Funding** [26](#_heading=h.2jxsxqh)

**11. Disclaimer** [26](#_heading=h.3j2qqm3)

**Guidelines for developing an open mode online mental health screening tool** [27](#_heading=h.1y810tw)

**1.** **Purpose or scope** [27](#_heading=h.4i7ojhp)

**2.** **Modes of testing** [27](#_heading=h.2xcytpi)

**3.** **Psychometric properties** [27](#_heading=h.1ci93xb)

**4.** **Informed consent** [29](#_heading=h.2bn6wsx)

**4.** **Ensuring minimal risk to participants** [30](#_heading=h.qsh70q)

**6.** **Feedback** [30](#_heading=h.3as4poj)

**7.** **Data Security** [31](#_heading=h.1pxezwc)

**References** [32](#_heading=h.49x2ik5)

# **Glossary of terms**

**Computerised testing:** tests are both administered and scored using any mobile device ranging from computers to phones or tablets (Davies, Foxcroft, Griessel & Tredoux, 2013).

**Mode of testing:** Modes of tests are set characteristics that distinguish various types of psychological tests by considering the following criteria: (1) administration conditions, (2) level of access and (3) level of supervision required. Hence there are four modes of testing:

- **Open tests** are available to any individual accessing the internet and requires no supervision. Therefore, the environmental conditions of testing cannot be standardised or guaranteed.
- **Controlled tests** are available to test–takers who are known. Therefore, a unique username and login detail is required for each individual test-taker. These details are often sent to the test-taker via email. There is no supervision required and the test-taker can take the test at a time and place of his or her own choice.
- **Supervised tests** require an administrator to login for the test-taker and confirm that test administration requirements are met thus allowing the test-taking condition to be controlled through some level of direct supervision. This mode also allows one to validate the identity of the test-taker.
- **Managed tests** are mostly administered in testing centres where a high level of supervision and control over the test taking conditions is required.

(APS Online Testing Guidelines, 2018; Bartram 2006; ITC International Guidelines on Computer-based and Internet Delivered Testing, 2005).

**Online testing**: Online psychological tests refer to a psychological test that is delivered on the internet where the test administration is located on a server found on the internet and not on the computer where the test is administered (Tredoux, 2019). The test-taker submits their responses via a keyboard, mouse or touch input and test results and feedback can either be sent directly to the test-taker or to the registered professional (Barak & Buchanan, 2004).

**Psychological assessment:** refers to the integration of the results , by an appropriately trained professional, of the results obtained with psychological measures and other information to make an assessment and inform conclusions - a single test does not constitute a full psychological assessment (Foxcroft & Roodt, 2013).

**Psychological testing and testing instruments** refer to questionnaires, tests, apparati, techniques or instruments which measure psychopathology, psychophysiological functioning, personality make-up or functioning, aptitude as well as intellectual abilities (Health Professions Act, No. 56 of 1974). Such instruments would usually not be conducted online, but are managed and administered personally by a psychological practitioner in a highly supervised test centre (Health Professions Act, No. 56 of 1974).

# **Screening tool/ test or instrument**: refers to a test or instrument (usually a questionnaire), which may be administered online, and which has been validated in certain populations to screen for the possible presence of a specific mental health condition. **It cannot provide a diagnosis.**

# **Introduction**

Over the past decade the internet has become more readily accessible, therefore, online mental health care has been proposed in low resource countries and communities as a means of increasing access to care. This has seen the development of many ethical guidelines for professionals working in this field. These documents were found to address the broad domains of professional and client interaction, psychological testing as well as e-therapy. However, there has been no specific set of guidelines aimed at the developers and professionals developing or utilising online screening tools for mental health. It is imperative in designing online screening tools that the ethical aspects relating to this aspect of mental health assessment be addressed.

# **Aim**

The aim of this document is to provide guidelines with regards to ethical considerations for all stakeholders involved in online mental health screening.

#

# **Objectives**

- To provide best practise guidelines with regards to screening instruments/tools (open mode tests) when being designed for online use or for research purposes
- To assist in ensuring that online mental health screening tools adhere to standard ethical guidelines.

This document should be read in conjunction with documents addressing international best practice guidelines and criteria linked to internet testing, particularly the International Test Commission (ITC) Guidelines on Test Use (ITC, 2013), the ITC Guidelines for Translating and Adapting Tests – Second Edition (ITC, 2017) and the ITC Guidelines on Computer-Based and Internet Delivered Testing (ITC, 2013).

These guidelines were originally developed to aid mental health screening in South Africa. Hence developers and practitioners should use this document in conjunction with the ethical rules of conduct for practitioners registered under the South African Health Professions Act, 1974 and the Mental Health Care Act, 2002 if being used in South Africa. For use outside of South Africa, the appropriate legislation or professional practice documents must be consulted alongside these guidelines.

# **Who are the guidelines for?**

The guidelines are applicable to the following group/s of individuals:

- Mental health test developers, publishers and consultants
- Professionals (eg. psychiatrists, psychologists, psychometrists, social workers and other allied healthcare professionals) wanting to utilise online mental health screening tests for their practice / for obtaining additional information or for research purposes
- Organisations utilising online mental health assessments on their websites

Students training in the field (eg. psychiatry, psychology, psychometry, social work or other allied healthcare professions)

Any individual mentioned above who will publish, use or design an online mental health screening tool should refer to this document as a guideline to facilitate best practise.

# **Process of Guideline development**

The development of these guidelines followed six phases as indicated in Figure 1. The first phase involved a systematic review of available literature to determine what was available in terms of guidelines. The first draft was compiled based on the guidelines located across the literature. The first draft was circulated to experts for comment using a qualitative design. The second phase involved a quantitative design, were experts utilised the Appraisal of Guidelines for Research and Evaluation to assess the quality of the guidelines. The phases are described in detail below.


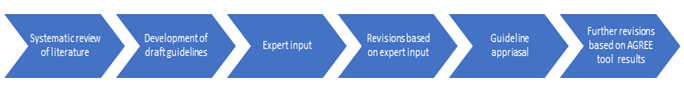


*Figure 1: Guideline development process*

## **Systematic review**

A systematic review was conducted to explore existing guidelines on online screening with a view to formulating recommendations for a more universally applicable standard for online screening of mental health (see Hassem & Laher, 2020). Articles were included in the review if the following two criteria were met: Ethical considerations for online screening or assessment were discussed (in relation to diagnosis or screening), and articles were published in English between 1970 to 2019 (this time frame was used as online psychological testing dates back to the early 1970s). Articles were excluded if there was no reference made to online/internet testing, screening or assessment. Articles that referred to another source in relation to online testing were excluded, but the original source was obtained and subjected to the inclusion and exclusion criteria. Results of the systematic review indicated that there was no single guideline document focussing on the ethics of online mental health screening. Rather broad guidelines alluding to screening in other more generic online ethical documents in the field of mental health were found.

## **Development of draft guidelines**

The draft guidelines were developed using the results of the systematic review as well as information from other guideline documents that addressed the online screening of mental. The following documents were the most influential in the development of this guideline:

Australian Psychological Society (2018). *Online Psychological testing*. Retrieved 22 January, 2019, from <https://www.psychology.org.au/APS/media/Resource-Finder/Testing/Online-psychological-testing.pdf>

Barak, A., & Buchanan, T. (2004). Internet-based psychological testing and assessment. In R. Kraus, J. S. Zack, & G. Stricker (Eds.), *Online counseling: A handbook for mental health professionals* (pp. 217–239). New York, NY: Elsevier Science. (2004-00189-011).

Bartram, D. (2006). The Internationalization of Testing and New Models of Test Delivery on the Internet. *International Journal of Testing*, *6*(2), 121–131. https://doi.org/10.1207/s15327574ijt0602_2

Buchanan, T. (2002). Online assessment: Desirable or dangerous? *Professional Psychology: Research and Practice*, *33*(2), 148–154. https://doi.org/10.1037/0735-7028.33.2.148

Evans, D. J. (2018). Some guidelines for telepsychology in South Africa. *South African Journal of Psychology*, *48*(2), 166–170. https://doi.org/10.1177/0081246318757943

Fisher, C. B., & Fried, A. L. (2008). Internet-mediated psychological services and the American Psychological Association Ethics Code. In D. N. Bersoff (Ed.), *Ethical conflicts in psychology., 4th ed.* (pp. 376–383). Washington, DC: American Psychological Association (2008-05541-084).

International Test Commission (2005). International guidelines on computer-based and internet delivered testing. International Journal of Testing, 6(2), 143-172. Retrieved 22 January, 2019, from http://www.intestcom.org/files/guideline_computer_based_testing.pdf

Joint Task Force for the Development of Telepsychology Guidelines for Psychologists. (2013). Guidelines for the practice of telepsychology. *American Psychologist*, *68*(9), 791–800. <https://doi.org/10.1037/a0035001>

Kier, F. J., & Molinari, V. (2004). Do-It-Yourself Testing for Mental Illness: Ethical Issues, Concerns, and Recommendations. *Professional Psychology: Research and Practice*, *35*(3), 261. https://doi.org/10.1037/0735-7028.35.3.261

Luxton, D. D., Nelson, E.L., & Maheu, M. M. (2016). Conducting psychological assessments during telemental health. In *A practitioner’s guide to telemental health: How to conduct legal, ethical, and evidence-based telepractice* (pp. 85–95). https://doi.org/10.1037/14938-008

Naglieri, J. A., Drasgow, F., Schmit, M., Handler, L., Prifitera, A., Margolis, A., & Velasquez, R. (2008). Psychological testing on the Internet: New problems, old issues. In D. N. Bersoff (Ed.), *Ethical conflicts in psychology, 4th ed.* (pp. 306–312). Washington, DC: American Psychological Association (2008-05541-067).

## **Expert input**

Once the first draft of the guidelines was developed, various international and national experts were invited to comment on the appropriateness of the guidelines. Each expert was provided with a working document of the guidelines and asked to provide feedback on the content, formatting or other issues using track changes and comments on the document. Experts were provided with a month to respond to the call. A total of 15 experts out of 51 approached responded and provided feedback regarding the document. The expert group consisted of psychiatrists, psychologists as well as experts in the field of psychological testing.

## **Revision**

Based on the results of the expert feedback received the draft guideline document was revised further and circulated to a second expert group for feedback regarding the guidelines based on the Appraisal of Guidelines for Research and Evaluation (AGREE) checklist (Brouwers, Kerkvliet, Spithoff, & AGREE Next Steps Consortium, 2016).

## **Guideline appraisal**

The revised guidelines together with the AGREE checklist was circulated to 31 international and local experts and 10 experts responded. The expert group consisted of psychologists and experts in the field of assessment. The AGREE checklist assessed the guidelines based on six broad domains; scope and purpose, stakeholder involvement, rigour of development, clarity of presentation, applicability and editorial Independence. Experts completed the checklist and also provided feedback for improvements on the guidelines using comments and track changes.

## **Revisions**

Based on the feedback provided on the AGEE checklist and recommendations made for improving the guidelines, the guidelines were further revised and presented in this document.

## **Procedures for updating the guidelines**

Guidelines will be updated every 5 years following the recommendations of the National Institute for Health Care Excellence (NICE) for updating guidelines (see: <https://www.nice.org.uk/process/pmg20/chapter/ensuring-that-published-guidelines-are-current-and-accurate>). For comments or recommendations on enhancing these guidelines email [Tasneem.hassem@wits.ac.za](mailto:Tasneem.hassem@wits.ac.za) or [Sumaya.Laher@wits.ac.za](mailto:Sumaya.Laher@wits.ac.za)

# **8. Acknowledgements**

The authors would like to thank the individuals who have provided valuable feedback during the drafting phases of these guidelines. This group consists of health practitioners in the fields of psychiatry, psychology, psychometrics and psychological assessment as well as experts in the field of online assessment. The authors express their appreciation to:

- Dr. Angelo Fynn - University of South Africa, South Africa
- Prof. Bernard Janse Van Rensburg - University of the Witwatersrand, South Africa
- Prof. Brendon Barnes - University of Johannesburg, South Africa
- Dr Cas Prinsloo - University of Pretoria & Assessment Standards South Africa
- Prof David Maree - University of Pretoria, South Africa
- Prof. Dragos Iliescu - University of Bucharest, Romania
- Mr. Dylan Evans - Clinical Psychologist, South Africa
- Dr Erica Munnik - University of Western Cape, South Africa
- Prof. Gabriel Ivbijaro - NOVA University, Portugal
- Prof. Graham Thornicroft - Institute of Psychiatry, Psychology and Neuroscience, King’s College, United Kingdom
- Prof. John Parker - University of Cape Town, South Africa
- Prof. Kate Cockcroft - University of the Witwatersrand, South Africa
- Prof. Lesley Robertson - University of the Witwatersrand, South Africa
- Dr. Mandy Wigdorowitz - University of Cambridge, St John’s College, United Kingdom
- Prof. Marie De Beer - M & M Initiatives, South Africa
- Ms. Nabeelah Bemath - University of the Witwatersrand, South Africa
- Dr. Nicola Taylor - JvR Psychometrics, South Africa
- Dr. Sherianne Kramer - School of Public Health, University of the Witwatersrand & Mental Health Unit (MHE), Department of Mental Health and Substance Use, World Health Organization (WHO), Netherlands
- Prof. Soraya Seedat - University of Stellenbosch, South Africa;
- Mrs. Ruby Patel - University of Witwatersrand, South Africa

# **9. Competing interests**

The authors declare that they have no competing interests.

# **10. Funding**

# This work is based on the research supported in part by the National Research Foundation of South Africa (Grant Number:112948).

#

# **11. Disclaimer**

The views and opinions expressed in this guideline are those of the authors and do not necessarily reflect the official policy or position of any affiliated agency of the authors.

# **Guidelines for developing an open mode online mental health screening tool**

The following guidelines are specific to development of an open mode online mental health screening tool and consists of seven sections, namely: (1) Purpose and scope (2) Modes of testing, (3) Psychometric properties, (4) Informed consent, (5) Ensuring minimal risk to participants, (6) Feedback and (7) Data security.

## **Purpose or scope**

- 1. Information regarding the construct being assessed as well as the aim and purpose of the screening instrument needs to be provided. The context in which results should be considered and the target population needs to be described

## **Modes of testing**

- 1. There needs to be a clear understanding and explanation of the type and mode of the screening test, which could be either open or controlled. For example, if the screening instrument is open mode then this should be clarified, that is, that the instrument is available to any individual on the internet and requires no supervision, while for a controlled screening instrument, it should be clarified that a unique user name is required (refer to Glossary of terms).

## **Psychometric properties**

Statements should be provided regarding:

- 1. The rigorous procedure which was followed for the development of the instrument, supported by the relevant psychometric information must be provided. For example, information pertaining to the reliability and validity of the instrument, and the sensitivity and specificity of the tool need to be stated amongst others.
  2. The relevance of the populations for which the instrument has been found to be reliable and/or valid should be discussed in relation to the individuals/groups to be tested as well as any limitations of the instrument.
  3. The user interface (tool layout and appearance) needs to be consistent across various devices. This needs to be checked and verified for usage across various devices (computers, mobile devices and tablets) in order to ensure accuracy of results and feedback. Where necessary, provision should be made for special populations, e.g. people with visual, auditory, or gross motor challenges.
  4. The user connectivity and data usage needs to be considered. Particular attention needs to be given to the data usage costs when designing a tool for low resource communities. Connection disruptions need to be programmed into the tool design in order for the user to continue with the test if interrupted due to connectivity issues.
  5. As screening instruments are typically for community members to use, many of the recommended testing conditions cannot be adhered to at the same level as controlled tests, e.g. appropriate time for taking the test; conditions under which the test should be taken - a quiet room that is well lit and also free from distractions such as friends, family, pets, loud noises, phones, etc. Hence:
     1. A brief description of the ideal test taking conditions need to be presented to the test taker. This should include but should not be limited to:
- Completing the test at a time when the test-taker will not be distracted or interrupted
- Completing the test when they are well rested and able to focus on the questions / statements and provide accurate responses
- The conditions in which they will be taking the test should ideally be well lit and quiet to ensure focus in responding as accurately as possible to the questions posed.
  - 1. The ideal testing conditions noted above are not likely to be available for low resource communities, however it is still recommended that they be included to the extent that the best possible space for the individual completing the screening can be available.
    2. A detailed description of what the test would require of the test-taker should be provided. Typically, this could include:
- What the test screens for
- Estimated time it will typically take for the test to be completed
- The manner in which responses should be completed in terms of the response format, ideal conditions, etc as in 3.5.1 and 3.5.2.
- A statement regarding the adherence to instructions in order to ensure that the most reliable and accurate test results can be obtained.
- What feedback can be expected, what resources can be accessed and who can be contacted for any questions that the test taker may have.

## **Informed consent**

- 1. In order to obtain consent participants should be provided with information regarding the process and this should be followed by a check box or similar mechanism. Consent is valid if done by checking a single checkbox or alternatively by checking several checkboxes. Failure to do so should prevent an individual from continuing to the next page.
  2. Information provided for consent purposes should state the following:
- The nature/purpose of the test;
- Who the target populations of the test are (e.g. country specific or international, age, if gender specific, specific nationality, religious or cultural group);
- The manner in which results will be obtained (see glossary of terms for information specific to the test mode);
- The open access nature of the screening (e.g. the tool can be accessed by anyone, does not require any form of supervision by a trained professional and scores are not directly interpreted by a trained professional);
- The manner in which results and information provided will be used (e.g. third party data sharing, data used for research purposes, any relevant legislation);
- Data storage (e.g. all responses will be stored on a cloud or a password protected folder)
- The possibility of false positives must also be clearly explained in terms of the test being a screening not a diagnosis and that results may not be accurate;
- Contact details for further information should be provided;
- The limitations of a screening test must be explained;
- Any risks to taking the test need to be specified;
- The test can be stopped at anytime or stage if needed - there should be no obligation to complete the test
- Appropriate referral sources should be provided
  1. In relation to limitations, the test-taker should be aware that screening has certain limitations; that screening represents a provisional process based on the information provided and cannot replace a formal diagnostic process and confirmation can only be done by an appropriately trained healthcare professional. The screening is just the first step in a longer process of seeking psychological help.

4.4. If there are risks to confidentiality in terms of the test data, results and feedback, these should be explained as well as the steps taken by the developers to prevent this from occurring. Confidentially of data should adhere to the relevant legislation of the country. for example, the Protection of Personal Information (POPI) Act will apply in South Africa. In the Europe the General Data Protection Regulation (GDPR) will apply. In addition, if the data is shared with third parties, a statement stating who will access the data and how the data will be utilised by the third party needs to be provided as well as obtaining user consent. **If** **the guidelines are used outside of South Africa, the relevant legislation for that particular country should be applied.**

4.5. The information provided should be given in a clear and easy-to-understand language (possibly in more than one language as specific to the population using the instrument) and should be free from field specific jargon.

## **Ensuring minimal risk to participants**

- 1. As instruments are open mode (accessible by anyone on the internet), they should have minimal risk to the individual.
  2. Basic information regarding seeking assistance with the relevant contact details should be present on every screen of the test as well as on the home page, consent page and the feedback page as an individual could feel overwhelmed at any point while taking the test.
  3. If potentially disturbing questions are asked, a red flag (or similar) warning should appear prior to the question
  4. Warnings need to be provided where limited access to mental health resources are available
  5. Flagging that mental health is treatable is highly recommended.
  6. Be cognisant of the potential impact of a mental health screening for the individual particularly in terms of self or public stigma that may be associated with a particular mental illness

## **Feedback**

- 1. Feedback for online screening instruments should be provided in clear and easy-to-understand language, informative but tentative, so that the potential negative effects of receiving a result pertaining to mental health are somewhat controlled. In addition, appropriate use of the results should be indicated. The possibility of a false-positive or false-negative test result should also be addressed. The nature of self-report measures of the screening tool should be explained.
  2. Typically, feedback should emphasise support that is available and should refer the test-taker to various psychological services that would be accessible to test-takers and which have been established to be reliable and accessible ideally at no cost. The referral should be accompanied with contact numbers, website addresses, online therapeutic tools and preferably include services provided by local clinics.
  3. In cases where the screening instrument does detect a great potential for self- harm, a red flag response with a possible action list should appear. This may include:
- Asking test-takers to provide a contact number or information where s/he can be reached if this is a possibility. If follow up is promised, it is imperative that follow-up is done. It could be an automated process to provide such information to pre-arranged reliable psychological services. If the assessment is completed anonymously, this cannot be undertaken – see data security below.)
- Alerting test-takers to pre-arranged reliable psychological services and/or information for a self-help service where someone will contact him/her to make and confirm an appointment – either by phone or online or in person is recommended.
- Encouraging test-takers to access available support services and where possible the local clinic for follow-up assistance is vital if indivdual follow up is not possible.

## **Data Security**

In order to ensure data security, the website should be secure to minimise the possibility of hacking. Generally screening instruments are completed anonymously, hence there should be no user information collected other than location, search terms, IP addresses, and so forth that are routinely collected on the “back-end” of a website. However, the website needs to keep this information safe and confidential. Routine backups should be done on the server. In order to ensure confidentiality and security of data one should adhere to the relevant legislation of a particular country. In South Africa this would be the POPI Act. **If the guidelines are used outside of South Africa, the relevant legislation for that particular country should be applied**

# **References**

Australian Psychological Society (2018). *Online Psychological testing*. Retrieved 22 January, 2019, from <https://www.psychology.org.au/APS/media/Resource-Finder/Testing/Online-psychological-testing.pdf>

Barak, A., & Buchanan, T. (2004). Internet-based psychological testing and assessment. In R. Kraus, J. S. Zack, & G. Stricker (Eds.), *Online counseling:  A handbook for mental health professionals.* (pp. 217–239). New York, NY: Elsevier Science. (2004-00189-011).

Bartram, D. (2006). The Internationalization of Testing and New Models of Test Delivery on the Internet. *International Journal of Testing*, *6*(2), 121–131. https://doi.org/10.1207/s15327574ijt0602_2

Brouwers, M. C., Kerkvliet, K., Spithoff, K., & AGREE Next Steps Consortium. (2016). The AGREE Reporting Checklist: a tool to improve reporting of clinical practice guidelines. *Bmj*, *352*, i1152.

Chipise, E. M., Wassenaar, D., & Wilkinson, A. (2018). Towards new ethics guidelines: the ethics of online therapy in South Africa. *South African Journal of Psychology*, 0081246318811562.

Davies, C., Foxcroft, C., Griessel, L., & Tredoux. N. (2013). Computer-based and internet-delivered assessment. In C. Foxcroft, & G. Roodt (Eds.), *Introduction to psychological assessment in the South African context. (4th Edition). Cape Town: Oxford University Press. (ISBN 978-0-1990-4473-3)*.

Foxcroft, C. D. (2004). Planning a psychological test in the multicultural South African context. *SA Journal of Industrial Psychology*, *30*(4), 8-15.

Foxcroft, C., & Roodt, G. (2019). An overview of assessment: definition and scope. In C. Foxcroft, & G. Roodt (Eds.), *Introduction to psychological assessment in the South African context. (5th Edition)* (pp.2-7). Cape Town: Oxford University Press. (ISBN 9780190418595).

Hassem, T., & Laher, S. (2020). The ethics of online screening for mental health in South Africa: A systematic review. *International Journal of Mental Health*, 1-17.

International Test Commission (2001). International Guidelines for Test Use, International Journal of Testing, 1(2), 93-114. Retrieved 10 May, 2020 from

<https://www.intestcom.org/files/guideline_test_use.pdf>

International Test Commission (2013). International guidelines on computer-based and internet delivered testing. International Journal of Testing, 6(2), 143-172. Retrieved 22 January, 2019, from <http://www.intestcom.org/files/guideline_computer_based_testing.pdf>

International Test Commission. (2017). The ITC Guidelines for Translating and Adapting Tests (Second edition). [www.InTestCom.org] Retrieved 10 May, 2020 from

<https://www.intestcom.org/files/guideline_test_adaptation_2ed.pdf>

Tredoux, N. (2019). Computer-based and internet-delivered assessment. In C. Foxcroft, & G. Roodt (Eds.), *Introduction to psychological assessment in the South African context. (5th Edition)* (pp.2-7). Cape Town: Oxford University Press. (ISBN 9780190418595).
